# Mechanisms of NMDA Receptor Inhibition by Sepimostat—Comparison with Nafamostat and Diarylamidine Compounds

**DOI:** 10.3390/ijms242115685

**Published:** 2023-10-27

**Authors:** Arseniy S. Zhigulin, Oleg I. Barygin

**Affiliations:** Sechenov Institute of Evolutionary Physiology and Biochemistry of RAS, 44, Toreza Prospekt, 194223 Saint Petersburg, Russia; arseniy.zhigulin@yandex.ru

**Keywords:** NMDA receptors, pharmacological modulation, patch clamp, sepimostat, nafamostat, gabexate, pentamidine, diminazene, guanidines, diarylamidines

## Abstract

N-methyl-D-aspartate (NMDA) receptors are inhibited by many amidine and guanidine compounds. In this work, we studied the mechanisms of their inhibition by sepimostat—an amidine-containing serine protease inhibitor with neuroprotective properties. Sepimostat inhibited native NMDA receptors in rat hippocampal CA1 pyramidal neurons with IC_50_ of 3.5 ± 0.3 µM at −80 mV holding voltage. It demonstrated complex voltage dependence with voltage-independent and voltage-dependent components, suggesting the presence of shallow and deep binding sites. At −80 mV holding voltage, the voltage-dependent component dominates, and we observed pronounced tail currents and overshoots evidencing a “foot-in-the-door” open channel block. At depolarized voltages, the voltage-independent inhibition by sepimostat was significantly attenuated by the increase of agonist concentration. However, the voltage-independent inhibition was non-competitive. We further compared the mechanisms of the action of sepimostat with those of structurally-related amidine and guanidine compounds—nafamostat, gabexate, furamidine, pentamidine, diminazene, and DAPI—investigated previously. The action of all these compounds can be described by the two-component mechanism. All compounds demonstrated similar affinity to the shallow site, which is responsible for the voltage-independent inhibition, with binding constants in the range of 3–30 µM. In contrast, affinities to the deep site differed dramatically, with nafamostat, furamidine, and pentamidine being much more active.

## 1. Introduction

The NMDA receptors are a subtype of ionotropic glutamate receptors, playing important roles in synaptic plasticity, learning and memory, neuronal development, and survival [1]. They are inhibited by many cationic compounds [2]. First, the voltage-dependent block with endogenous magnesium [3,4] determines many important NMDA receptor properties, including bell-shaped voltage dependence, allowing them to act as coincidence detectors. Second, many pharmaceuticals inhibit NMDA receptors by different mechanisms. For some of them, NMDA receptors are among the primary targets. Memantine—a moderate affinity NMDA receptor open channel blocker—is used in Alzheimer’s disease treatment [5]. Ketamine—another moderate affinity NMDA receptor open channel blocker—is used as a dissociative anesthetic and as a rapid-acting antidepressant [6,7]. Dextromethorphan—yet another NMDA receptor channel blocker—is an antitussive agent that was recently approved for treatment of major depressive disorder in combination with bupropion [8]. For many other pharmaceuticals, NMDA receptors are an additional target that can possibly mediate negative or positive side effects. For instance, they are inhibited by traditional antidepressants desipramine and fluoxetine [9,10], antihistamine compounds promethazine [11] and diphenhydramine [12], and local anesthetics bupivacaine, lidocaine, procaine, and tetracaine [13].

In addition, NMDA receptors are inhibited by many compounds, possessing amidine or guanidine groups. Antimicrobial diarylamidine medication pentamidine [14,15] is a potent NMDA receptor antagonist with an IC_50_ value in submicromolar range. In our previous papers [16,17], we have shown that this ability to inhibit NMDA receptors is shared by several other diarylamidine compounds—diminazene, furamidine, and DAPI. All these compounds are structurally similar to nafamostat—a serine protease inhibitor with one amidine and one guanidine group. Indeed, we have shown using patch clamping that nafamostat inhibits native NMDA receptors with an IC_50_ value of 0.20 ± 0.04 µM at −80 mV holding voltage [17]. Moreover, another serine protease inhibitor—gabexate—inhibited native NMDA receptors as well [17] while the third one—camostat—was practically ineffective [17]. Nafamostat acted as a “foot-in-the-door” NMDA receptor channel blocker. The action of gabexate was mostly voltage-independent and not competitive, suggesting allosteric NMDA receptor inhibition.

Sepimostat mesilate (FUT-187), an orally active derivative of nafamostat, is another amidine-containing inhibitor of serine proteases [18]. Both sepimostat and nafamostat demonstrated significant activity in the animal pancreatitis models [19,20]. However, only nafamostat is used for acute pancreatitis treatment in humans while development of sepimostat was discontinued for the unknown reasons. In addition, it has been shown recently that both compounds have retinoprotective properties [21]. Fuwa and coauthors [21] hypothesized that retinal protection with nafamostat and sepimostat is the consequence of GLUN2B-containing NMDA receptor inhibition because both compounds inhibited [3H]ifenprodil binding to fractionated rat brain membranes in micromolar concentrations with IC_50_ values of 4.52 and 29.8 µM, respectively. In contrast, gabexate and camostat—two other serine protease inhibitors—did not demonstrate retinal protection activities in their experiments.

In this paper, we used patch clamping to check whether sepimostat would be able to inhibit native NMDA receptors and to describe its kinetics and molecular mechanisms of action. We compared the mechanisms of NMDA receptor inhibition by sepimostat to those of nafamostat and gabexate [17], furamidine [17], pentamidine, diminazene, and DAPI [16]. All these compounds are somewhat structurally similar, having aromatic cycles in the central parts of the molecules and positively charged nitrogene-containing groups on at least one side. Our comparative analysis demonstrated that the action of all these compounds can be readily described by the two-component mechanism. All compounds demonstrated similar affinity to the shallow site, which is responsible for the voltage-independent inhibition with binding constants in the range of 3–30 µM. In contrast, affinities to the deep site, which corresponds to the open-channel block, differed dramatically, with nafamostat, furamidine, and pentamidine being much more active. Among the compounds, only flexible pentamidine demonstrated a trapping block, gabexate did not demonstrate any signs of a “foot-in-the-door” or trapping channel block, and all others were “foot-in-the-door” blockers.

## 2. Results

### 2.1. NMDA Receptor Inhibition by Sepimostat

#### 2.1.1. Concentration Dependence

**Figure 1:**
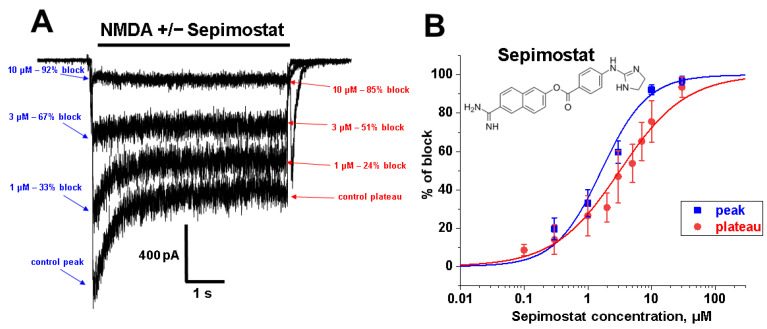
Concentration dependence of sepimostat action on NMDA receptors. (**A**) Representative examples of NMDA receptors inhibition by different concentrations of sepimostat. (**B**) Chemical structure of sepimostat and its concentration–inhibition curves for peak component (blue) and steady-state (plateau) component (red).

Application of extracellular solutions containing NMDA 100 µM and glycine 10 µM induced desensitizing inward currents in pyramidal cells of the CA1 region of the hippocampus with a steady-state component of about 300–1000 pA at −80 mV holding voltage. Different concentrations of sepimostat were applied simultaneously with agonists leading to a reversible concentration-dependent inhibition of the peak and steady-state component of the currents through NMDA receptors (Figure 1A). High concentrations of sepimostat virtually eliminated the peak component of the response. Fitting the data on concentration dependencies of the action on the peak and steady-state components with the Hill equation (Figure 1B) gave IC_50_ values and Hill coefficients of 1.8 ± 0.4 µM and 1.1 ± 0.2 (peak) and 3.5 ± 0.3 µM and 0.9 ± 0.1 (steady-state), respectively. The sensitivity of different CA1 pyramidal neurons to sepimostat was similar. It is worth noting that sepimostat was significantly less active than nafamostat (IC_50_ = 0.20 ± 0.04 µM) [17] in this experimental series.

#### 2.1.2. Voltage Dependence

**Figure 2:**
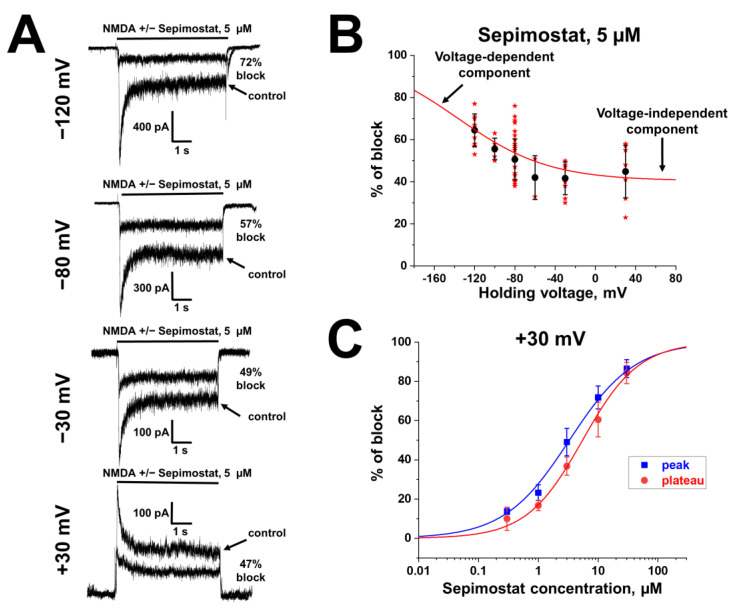
Voltage dependence of sepimostat action on NMDA receptors. (**A**) Representative traces of 5 µM sepimostat action at different holding voltages. (**B**) Summary of voltage dependence data for steady-state currents inhibition. The data are presented as the mean ± SD (black). Red curve demonstrates the fitting of pooled together experimental data (red asterisks) from Equation (2). Note: for −60 mV, some values of the block are identical. (**C**) Concentration–inhibition curves for peak component (blue) and steady-state (plateau) component (red) at +30 mV holding voltage.

A sepimostat molecule contains one amidine and one 4,5-dihydro-1H-imidazol-2-ylamino group (Figure 1B). Charged groups allow us to estimate the location of binding sites of the molecule in the membrane electrical field by analyzing the voltage dependence of action. We studied sepimostat in the range from −120 to +30 mV. Representative traces of 5 µM sepimostat action at different holding voltages are shown in Figure 2A. NMDA receptor inhibition by sepimostat demonstrated complex voltage dependence with voltage-independent and voltage-dependent components both for the peak and steady-state (Figure 2B) currents inhibition in contrast to that of nafamostat, which was strongly voltage-dependent [17]. Complex voltage dependence does not allow us to use the simplest equation to reveal the binding site location. An approximation with Equation (2) (see Section 4), which takes into account both voltage-dependent and voltage-independent components of action, gave zδ values of 0.7 ± 0.2 (peak) and 0.6 ± 0.2 (steady-state). Taking into account the similarity of voltage dependence for peak and steady-state components, in this work, we performed further analysis, mainly, for the steady-state one. According to Chemaxon (https://chemaxon.com/, accessed on 5 September 2023) predictions, a sepimostat molecule possesses a +1 charge predominantly (83.2%—monocation, 16.8%—dication) at pH 7.4. Thus, the δ value can be considered to be 0.6 ± 0.2 as well. The constant for voltage-independent binding (K_vi_) was 7 ± 1 µM, and the constant for voltage-dependent binding (K_vd_) was ~130 µM. Thus, sepimostat has much higher affinity for the external site than for the site in the channel pore. As a result, the voltage-dependent inhibition is seen only at hyperpolarized membrane potentials, which enhance the voltage-dependent action. The concentration–inhibition curves for sepimostat action at conditions favoring binding to the external site (+30 mV) are shown in Figure 2C. The IC_50_ values and Hill coefficients were 3.5 ± 0.8 µM and 0.9 ± 0.1 (peak) and 5.8 ± 1.5 µM and 0.9 ± 0.1 (steady-state), respectively. The IC_50_ value for the steady-state component (5.8 µM) is in good agreement with the K_vi_ value (7 µM) derived from the voltage dependence data analysis.

We performed further experiments to better understand the nature of both voltage-dependent and voltage-independent components of inhibition by sepimostat and other guanidine and diarylamidine compounds.

#### 2.1.3. Interaction with Channel Gate

According to interaction with channel gate the ion channel blockers are divided into two major classes. Trapping blockers can remain in the closed channels while “foot-in-the-door” compounds prevent either channel desensitization, channel closure, agonist dissociation, or all of these processes [22]. We have found earlier that among amidine compounds, both types of blockers are present. Indeed, pentamidine demonstrated partial trapping in NMDA receptor channels [16] while diminazene, DAPI [16], furamidine, and nafamostat [17] demonstrated a “foot-in-the-door” mechanism. Thus, we decided to determine whether sepimostat is a “foot-in-the-door” or trapping blocker.

**Figure 3:**
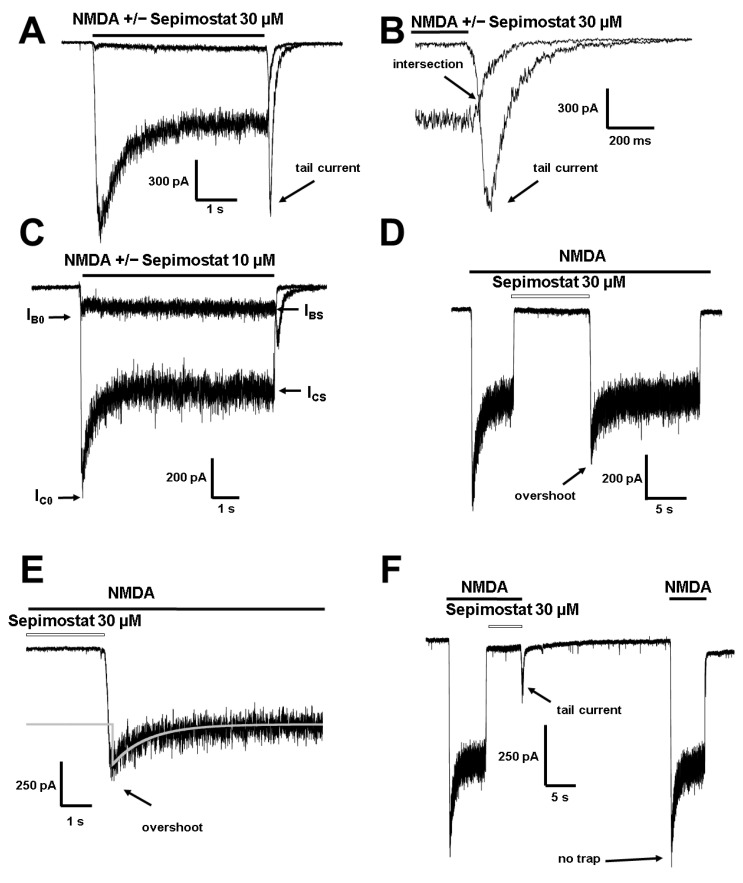
Interaction of sepimostat with channel gate of NMDA receptors. (**A**,**B**) Sepimostat, 30 µM demonstrates tail currents in case of washout in the absence of the agonists. The tail current after coapplication of sepimostat and agonists intersects with control tail current. Original protocol (**A**). Representation of tail current in more detail (**B**). (**C**) Sepimostat, 10 µM increases plateau/peak ratio. (**D**,**E**) Sepimostat, 30 µM demonstrates overshoots in case of washout in the presence of the agonists. Original protocol (**D**). Representation of overshoot in more detail (**E**). Kinetics of overshoot falling phase is well fitted by single exponential function (shown in light grey). (**F**) Sepimostat, 30 µM does not demonstrate trapping in “double-pulse” protocol.

In our experiments, sepimostat in high concentrations (30 µM, >90% block) at −80 mV holding voltage demonstrated tail currents that significantly prolonged the response (Figure 3A,B). The amplitude of the tail currents relative to the steady-state control response was 1.3 ± 0.5 (*n* = 6). The intersection of the control tail currents and the tail currents after the agonist and the blocker coapplication (Figure 3B) is a distinctive feature of “foot-in-the-door” blockers.

In a thorough paper, Sobolevsky and coauthors [22] elaborated on criteria that allow concluding about the effect of compounds on channel desensitization. The effect on channel desensitization can be determined by measuring the plateau/peak ratio in the absence and presence of the blocker and by analyzing the kinetics of the washout of the blocker in the presence of the agonists. In control conditions at −80 mV holding voltage, the plateau/peak ratio in our experiments was 0.6 ± 0.1 (Figure 3C, *n* = 4). For 10 µM sepimostat concentration, the plateau/peak ratio was significantly increased (1.1 ± 0.3, paired *t*-test, *p* < 0.05), implying that only nonblocked channels can desensitize. According to Sobolevsky and coauthors the normalized plateau/peak ratio ((I_BS_/I_B0_)/(I_CS_/I_C0_)) > 1 implies that the blocker prevents channel desensitization. This ratio was 1.8 ± 0.1 for 10 µM sepimostat (*n* = 4) at −80 mV holding voltage, suggesting the prevention of channel desensitization. At +30 mV holding voltage, the plateau/peak ratio in control was 0.52 ± 0.09 (*n* = 4), which was not significantly different from the plateau/peak ratio at −80 mV (0.6 ± 0.1). These data suggest that calcium-dependent inactivation does not play a significant role in the NMDA receptor desensitization in our experimental conditions and that the inhibitory action of sepimostat is not related to the effect on calcium-dependent inactivation.

Another sign of the prevention of desensitization by a channel blocker is the appearance of an overshoot after the removal of a blocker in the continuous presence of the agonists, and it was also observed in the case of 30 µM sepimostat (Figure 3D,E). Such an increase in the current amplitude is explained by a shift in the equilibrium of channel activation towards the open state. The amplitude of overshoot relative to the steady-state control response was 1.8 ± 0.3 (*n* = 4). The kinetics of the overshoot rising phase was very fast: τ_rise_ = 62 ± 15 ms (*n* = 4). That of the falling phase was significantly slower (τ_fall_ = 770 ± 140 ms, *n* = 4). It is worth mentioning that nafamostat did not significantly change the plateau/peak ratio and did not demonstrate overshoots, evidencing the absence of its effect on NMDA receptor desensitization, in contrast to sepimostat [17].

To fully exclude the trapping effect, we tested sepimostat in the “double-pulse” protocol [23,24], which consists of the control NMDA response, a deep block with 30 µM sepimostat, and a pause in the extracellular solution and testing NMDA response. In the case of trapping, the peak of the testing response is inhibited in comparison to the peak of the control response as blocker molecules remain in closed channels during the pause. As expected, there were no signs of trapping in our experiments (Figure 3F, *n* = 5).

#### 2.1.4. Absence of Competition with Magnesium for Binding Site

An analysis of the voltage-independent mechanism of block requires experimental conditions that minimize the voltage-dependent component. At low voltages, the currents are too small for a precise analysis; at positive voltages, the low clamp stability complicates long complex experiments. Therefore, we tested sepimostat action at −30 mV. It is worth noting that at −30 mV holding voltage, the amplitude of the tail currents was significantly smaller than at −80 mV holding voltage, and the tail currents after the agonist and the blocker co-application did not intersect with control tail currents.

One of the most important physiological properties of NMDA receptors is a voltage-dependent block by magnesium ions [3,4]. Because of the competition with magnesium ions for the binding site in the channel pore, the activity of many NMDA receptor channel blockers is significantly reduced [25,26]. In the presence of 1 mM magnesium, the current through NMDA receptors at −30 mV is close to maximal comparing to the currents at other holding voltages. In the case of competition, the presence of magnesium would reduce the drug activity. Activity of nafamostat, which causes voltage-dependent inhibition, in such an experiment was attenuated in the presence of magnesium, suggesting competition for the same binding site [17].

**Figure 4:**
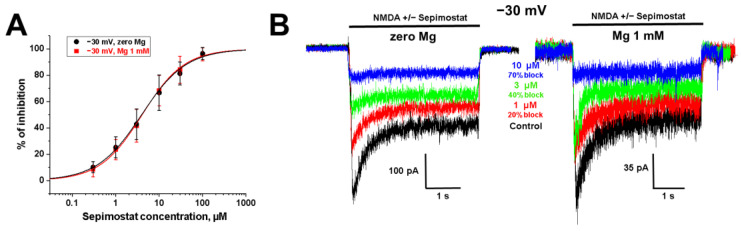
Sepimostat does not compete with magnesium ions for binding site in NMDA receptor channel. (**A**) Concentration dependencies of sepimostat action in the absence and presence of 1 mM Mg^2+^ at −30 mV holding voltage. (**B**) Representative examples of NMDA receptor inhibition by different concentrations of sepimostat in the presence and absence of 1 mM Mg^2+^ at −30 mV holding voltage.

The IC_50_ values for sepimostat action in the presence and absence of magnesium (Figure 4) were 3.6 ± 1.1 µM (*n* = 7) and 3.6 ± 1.0 µM (*n* = 5), respectively, evidencing the idea that sepimostat does not compete with Mg^2+^ for a binding site in the NMDA receptor channel pore at −30 mV holding voltage (*p* > 0.05, unpaired *t*-test). These data suggest the prevalence of a voltage-independent component of inhibition at −30 mV. It is worth mentioning that at −30 mV, in the presence of 1 mM magnesium ions, nafamostat was only two-fold more active, than sepimostat, with IC_50_ values 1.7 ± 0.2 and 3.6 ± 1.1 µM, respectively. The difference in activities at −80 mV in the absence of magnesium was ~20-fold (IC_50_ = 0.20 ± 0.04 µM for nafamostat and 3.5 ± 0.3 µM for sepimostat). These data once again emphasize the importance of measuring activities against NMDA receptors in conditions that are closer to physiological.

#### 2.1.5. Voltage-Independent Inhibition by Sepimostat Is Agonist-Dependent but Not Competitive

**Figure 5:**
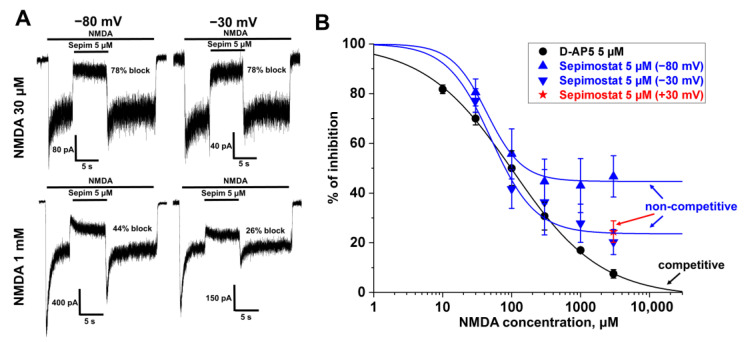
Agonist dependence of the sepimostat action on NMDA receptors. (**A**) Representative example of NMDA receptor inhibition by 5 µM sepimostat at two different NMDA concentrations (30 and 1000 µM) at −80 and −30 mV holding voltages. (**B**) Summary of the agonist dependence data for 5 µM sepimostat (blue and red) and 5 µM D-AP5 (black), a classical competitive NMDA receptor antagonist. The inhibition by D-AP5 at high NMDA concentrations became weak, decaying to zero, suggesting the competitive mechanism. The inhibition by sepimostat at high NMDA concentrations was attenuated at −30 mV and +30 mV holding voltages but remained significant, suggesting that voltage-independent binding of sepimostat is not competitive. The data are presented as the mean ± SD. The curves demonstrate the fitting of pooled together experimental data with the Hill equation.

To reveal the mechanism of the voltage-independent inhibition, we first checked if it depended on NMDA concentration. In our experiments, the percentage of inhibition by 5 µM sepimostat significantly decreased with the increase in NMDA concentration from 30 to 1000 µM (Figure 5A) at −80 mV holding voltage. Attenuation of the effect in case of higher agonist concentration is a typical feature of competitive antagonists. To fully exclude this possibility, we compared the agonist dependencies of the action of sepimostat and D-AP5, a classical competitive NMDA receptor inhibitor (Figure 5B). Sepimostat demonstrated strong inhibition (about 50%) even in the case of high (1000 and 3000 µM) NMDA concentrations, evidencing the idea that inhibition is not competitive. In contrast, inhibition by D-AP5 at high NMDA concentrations became weak, decaying to zero.

At −80 mV holding voltage, the action of sepimostat includes a voltage-dependent component. To avoid it, we performed an analogous analysis at −30 mV, where the voltage-independent action dominates (see above). The inhibition by sepimostat at these high NMDA concentrations was attenuated at −30 mV holding voltage but remained significant at high NMDA concentrations (Figure 5B), suggesting that the voltage-independent binding of sepimostat is not competitive. Finally, we checked the sepimostat action at 3 mM NMDA concentration and +30 mV holding voltage (25 ± 4% block, *n* = 4, Figure 5B). At this voltage, the effect of sepimostat did not differ from its action at −30 mV (20 ± 5% block, *n* = 10, *p* > 0.1, unpaired *t*-test).

### 2.2. Comparison of the Sepimostat Action Mechanisms with Those of Structurally Related Compounds

Sepimostat is structurally very similar to nafamostat, a “foot-in-the-door” NMDA receptor channel blocker described by us previously [17]. They both have (6-carbamimidoylnaphthalen-2-yl) benzoate cores, and the only difference in their structures is that nafamostat possesses a guanidine group while sepimostat—4,5-dihydro-1H-imidazol-2-ylamino one in the same place. In addition, nafamostat and sepimostat are somewhat structurally similar to two other serine protease inhibitors—gabexate and camostat [17]—and to diarylamidine compounds—furamidine [17], diminazene, pentamidine, and DAPI [16]. Indeed, all these compounds possess aromatic rings in the central parts of their molecules and amidine and/or guanidine groups on at least one side. The chemical structures are presented in Table 1. Our present results with sepimostat allow for a systematic comparison of the action of all these structurally related drugs.

**Figure 6:**
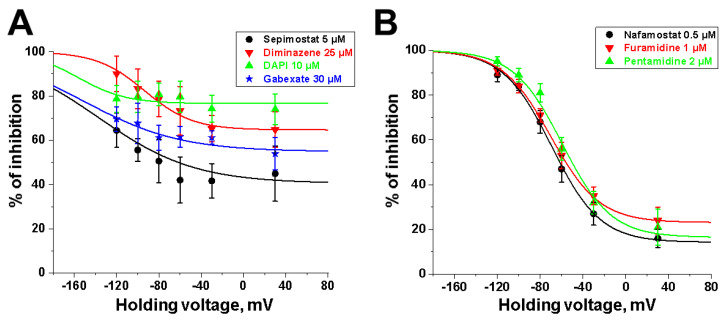
The comparison of voltage dependencies of compounds. (**A**) The action of sepimostat (5 µM), diminazene (25 µM), DAPI (10 µM), and gabexate (30 µM) is mostly voltage-independent. (**B**) The action of nafamostat (0.5 µM), furamidine (1 µM), and pentamidine (2 µM) is mostly voltage-dependent. The data are presented as the mean ± SD. The curves demonstrate the fitting of pooled-together experimental data by Equation (2).

NMDA receptor inhibition by sepimostat was mostly voltage-independent with a small voltage-dependent component. Among the compounds studied, a very similar voltage dependence was demonstrated by diminazene (Figure 6A). Completely voltage-independent inhibition was demonstrated by DAPI and gabexate (Figure 6A). In contrast, the action of three other compounds—nafamostat, furamidine, and pentamidine—was strongly voltage-dependent (Figure 6B). The use of Equation (2) allowed us to determine the binding constants to deep and shallow sites for sepimostat. We decided to reanalyze our data on the voltage-dependencies of the action of nafamostat, furamidine, pentamidine, diminazene, gabexate, and DAPI [16,17] using Equation (2) for the first time. The binding constants for the deep (K_vd_) and shallow (K_vi_) sites are presented in Table 1.

All parameters are very close for the voltage-dependent blockers nafamostat, furamidine, and pentamidine, including zδ values of 1.1 to 1.4. The second group includes sepimostat and diminazene with large K_vd_ values and zδ values of 0.6 and 0.9, respectively. For the voltage-independent inhibitors DAPI and gabexate, the parameters, which describe a voltage-dependent block, cannot be estimated reliably. However, the experimental data on gabexate and DAPI can be readily fitted with the consensus zδ value 1.1 for the doubly charged DAPI (taken from fitting of nafamostat and furamidine) and 0.55 for the single-charged gabexate (taken from fitting of sepimostat) (see Table 1 and Figure 6). According to this fitting, all compounds demonstrate the presence of a voltage-independent component of action. Moreover, the obtained binding constants to the shallow site all laid in the narrow range from 3 to 30 µM. The voltage-dependent component of the action is described by consensus zδ values of 0.55 for monocationic compounds and 1.1 for dicationic compounds. The binding constants for the deep site differed dramatically, ranging from 10 µM for nafamostat to >1 mM for sepimostat, diminazene, and DAPI. Thus, the main variations in the voltage dependence seen in Figure 6 are due to the difference in only one characteristic (K_vd_). The single exception is camostat, which exhibited low activity and was not studied in detail.

Finally, we compared the agonist dependence of the action of all the compounds. It has been shown previously that the voltage-independent inhibition by gabexate is agonist-dependent but non-competitive [17]. In the present work, we demonstrated that the voltage-independent component of the sepimostat action is also agonist-dependent but non-competitive. For other compounds, the agonist dependence was not tested previously. To complete the dataset, we compared the action of nafamostat, pentamidine, diminazene, furamidine, and DAPI at 30 and 1000 µM NMDA concentrations. For compounds with high voltage-dependent component of block (nafamostat, furamidine, and pentamidine), the inhibition of responses was not attenuated significantly with the increase in NMDA concentration. For diminazene, that demonstrates complex voltage dependence, the inhibition did not differ significantly at −80 mV (*n* = 5, *p* > 0.05, paired *t*-test), but at −30 mV, the significant attenuation was observed (*n* = 5, *p* < 0.01, paired *t*-test). The voltage-independent action of DAPI was found to be agonist-dependent at both −80 mV (*n* = 5, *p* < 0.01, paired *t*-test) and −30 mV (*n* = 5, *p* < 0.05, paired *t*-test). However, for both diminazene and DAPI, the inhibition effect at −30 mV remained significant at 1 mM NMDA concentration. Thus, we can conclude that for all compounds belonging to the structural family, the voltage-independent component of the action is agonist-dependent but non-competitive.

## 3. Discussion

In this paper, we have shown that sepimostat inhibits native rat hippocampal NMDA receptors by interacting with two sites—shallow and deep. It acts as a voltage-dependent “foot-in-the-door” NMDA receptor channel blocker and also causes voltage-independent inhibition, which is agonist-dependent but non-competitive. At −80 mV holding voltage, sepimostat inhibited peak currents stronger than plateau currents, which is typical for fast “foot-in-the-door” NMDA receptor channel blockers, for example, tetrapentylammonium [22]. NMDA receptors of CA1 pyramidal cells contain mainly GluN2A and/or GluN2B subunits in addition to GluN1 [27]. The presence of both GluN2B-containing and GluN2B-lacking NMDA receptors in CA1 pyramidal cells was also confirmed by us previously in experiments with the NR2B-selective antagonist ifenprodil [28]. Indeed, the concentration dependence for ifenprodil was clearly biphasic, with the high-affinity component corresponding to inhibition of GluN2B-containing NMDA receptors and the low-affinity component corresponding to inhibition of GluN2B-lacking receptors. The high-affinity component accounted for approximately 60% of the inhibition of NMDA receptor currents in CA1 pyramidal cells. In our current experiments, practically full (>90%) inhibition of NMDA receptor currents was achieved at a relatively low 10 μM sepimostat concentration and its concentration dependence was monophasic; thus, sepimostat is able to inhibit both GluN2B-containing and GluN2B-lacking NMDA receptors. These data suggest that two-component mechanism is not due to significant differences in the action of sepimostat on GluN2A and GluN2B NMDA receptors, though this possibility cannot be fully excluded.

We have also systematically compared molecular mechanisms of inhibition by sepimostat to those of other serine protease inhibitors (nafamostat, gabexate, and camostat) and diarylamidine compounds (pentamidine, furamidine, diminazene, and DAPI). Except for DAPI, abovementioned compounds demonstrated complex voltage dependence with voltage-dependent and voltage-independent components, suggesting binding to the deep site in the pore and a superficial site. The action of DAPI was completely voltage-independent. Likely, affinity for the deep binding site is low, and a weak voltage-dependent component of the action is masked by the voltage-independent inhibition. The analysis of voltage dependencies of nafamostat, furamidine, and diminazene gave zδ values of about 1.0. That of pentamidine was higher—1.4. This subtle difference can be easily explained by the flexibility of pentamidine molecule, resulting in the interaction of both charged groups with the selectivity filter [16]. In the case of nafamostat, furamidine, and diminazene, the first charged group interacts with the selectivity filter while the second one is situated shallower because of the rigidity of these molecules [16,17]. The zδ value for sepimostat was two times smaller (0.6), which is in a good agreement with the domination of its +1 charged form. The deep site for channel blockers (Mg^2+^, memantine, ketamine, MK-801, and others) in the NMDA receptor channel pore is well characterized [29,30]. Such compounds bind to the NMDA receptor selectivity filter asparagines. Their interactions with pore-lining residues are slightly different [30], but the binding region is the same. Our data suggest that the voltage-dependent component of the action of nafamostat, sepimostat, pentamidine, furamidine, and diminazene corresponds to the binding to this site.

The flexibility of pentamidine molecule also explains why only this compound from this structurally similar group is able to stay in the closed NMDA receptor channel [16]. The molecule can fold into the compact form, which fits the cavity of the closed channel between the selectivity filter and the extracellular gate. “Foot-in-the-door” NMDA receptor antagonists from this group—sepimostat, nafamostat, diminazene, and DAPI—all have a rigid elongated structure and prevent channel closure. The voltage-dependent action of these compounds resembles the action of dicationic adamantane derivatives with bulk terminal groups [31]. They cannot bypass the tight selectivity filter and bind between it and the gate region. In such a binding pose, the long molecules sterically prevent the pore closure.

The location of the superficial site is not so clear. Our data suggest that the action significantly decreases with the rise in NMDA concentration, but it is non-competitive. Among non-competitive antagonists, such a decrease in activity with the increase in agonist concentration has been shown earlier for the NMDA receptor “foot-in-the-door” blocker tetrabutylammonium [22]. It is important to note that according to our data, all compounds demonstrate similar binding affinity to this superficial site, but their binding to deep site is drastically different (Table 1). It is that drastic difference that determines the diversity of the total action.

Different types of NMDA receptor antagonists differentially affect synaptic transmission in different conditions. The action of voltage-dependent channel blockers, including nafamostat, furamidine, and pentamidine, will be attenuated with depolarization, which often occurs in the case of pathological glutamatergic excitotoxicity. However, because of their ability to bind to a superficial site, the decrease in activity will not be as strong as for classical blockers without such an ability. The activity of mostly voltage-independent compounds, including sepimostat, diminazene, and DAPI, will not be affected by depolarization practically. In addition, “foot-in-the-door” and trapping channel blockers differentially affect synaptic activity with different frequencies. “Foot-in-the-door” blockers will equipotently inhibit each post-synaptic excitatory potential. In contrast, trapping blockers will accumulate in the closed channels, resulting in a stronger inhibition of high frequency activity. For evaluating possible therapeutic efficiency and safety, both activities, kinetics and mechanisms of action of the drug, should be taken into account. For instance, the favourable clinical profile of memantine, an NMDA receptor channel blocker used for Alzheimer’s disease treatment, is associated with its moderate affinity, rather fast kinetics, and partial trapping [5]. At −30 mV holding voltage in the presence of 1 mM magnesium ions, nafamostat (IC_50_ = 1.7 ± 0.2 µM) and sepimostat (3.6 ± 1.1 µM) are even slightly more active than memantine (IC_50_ = 6.4 ± 0.5 µM, [26]). Both sepimostat and nafamostat have rather fast washout kinetics. Thus, the appearance of negative side effects due to a prolonged NMDA receptor block by these compounds seems unlikely. With this in mind, nafamostat and sepimostat can be viewed as potential candidates for the treatment of neurodegenerative diseases associated with NMDA receptor overactivation, including Alzheimer’s disease and glaucoma.

## 4. Materials and Methods

### 4.1. Animals

All experimental procedures were approved by the Animal Care and Use Committee of the Sechenov Institute of Evolutionary Physiology and Biochemistry of the Russian Academy of Sciences (protocol 1-2/2022, 27 January 2022). Outbred male Wistar rats (13–18 days old and weighing 25–35 g) were obtained from a local (IEPHB) facility. Maximum efforts were made to minimize the number of animals used and to minimize discomfort.

### 4.2. Electrophysiology

The rats were anesthetised with sevoflurane and then decapitated. The brains were brought out quickly and cooled to 2–4 °C. Transverse hippocampal slices were cut using a vibratome (Campden Instruments Ltd., Loughborough, UK) and stored in a solution containing (in mM): NaCl 124, KCl 5, CaCl_2_ 1.3, MgCl_2_ 2.0, NaHCO_3_ 26, NaH_2_PO_4_ 1.24, D-glucose 10, aerated with carbogen (95% O_2_, 5%CO_2_). All experiments were performed at room temperature.

Vibrodissociation method [32,33] was used to free CA1 pyramidal neurons from slices. This method allows for isolating cells without enzymatic treatment and keeping them in more native state. Pyramidal cells were isolated from the stratum pyramidale and distinguished from non-pyramidal cells on the basis of pyramidal-like somata and preserved apical dendrites. In addition, the kainate-induced currents in CA1 pyramidal cells are virtually insensitive to calcium-permeable AMPA receptor channel blocker IEM-1460 in contrast to hippocampal interneurons [34], and that was used as additional pharmacological criterion in our experiments.

To record membrane currents in response to applications of NMDA and glycine, the whole-cell configuration of patch clamp technique was used. Series resistance (<20 MΩ) was compensated by 70–80% and monitored during experiments. Only cells with stable holding currents were used in further analysis. The current signals were amplified using EPC-8 (HEKA Electronics, Lambrecht, Germany), filtered at 5 kHz, sampled, and stored on a personal computer. RSC-200 (BioLogic Science Instruments, Claix, France) perfusion system was used to apply the drugs under computer control. The composition of extracellular solution (in mM) was as follows: NaCl 143, KCl 5, CaCl_2_ 2.5, D-glucose 18, HEPES 10 (pH adjusted to 7.4 with HCl). The pipettes with resistance of 2–5 MΩ were filled with the following solution (in mM): CsF 100, CsCl 40, NaCl 5, CaCl_2_ 0.5, EGTA 5, HEPES 10 (pH adjusted to 7.2 with CsOH). Sepimostat (HY-136299) was obtained from MedChemExpress (Monmouth Junction, NJ, USA). Other reagents were purchased from MedChemExpress (Monmouth Junction, NJ, USA), Sigma (St. Louis, MO, USA), or Tocris Bioscience (Bristol, UK).

NMDA receptors were activated with 100 µM NMDA plus 10 µM glycine unless otherwise stated. The percentages of blocking of the steady-state or peak currents by different drug concentrations were measured at −80, −30 or +30 mV holding voltages. Kinetics of transient processes of more than 20 ms duration were approximated with single or double exponential functions. In case of double exponential fitting, the weighted time constant was used.

### 4.3. Analysis of Voltage Dependence

The voltage dependence of compounds’ action was analyzed with the classical Woodhull model [35] with the addition of a voltage-independent component. According to the Woodhull model of an impermeable blocker, the voltage dependence of steady-state blockade is given by Equation (1):

(1) B=100/(1+Kb/C∗exp⁡〖Fzδ/RTV〗),

where V is voltage, B is level of block (%), C is concentration of the drug, z is molecular charge, and R, F, and T have their standard meanings. Kb is the affinity of a drug for the channel, and δ is the electrical depth of the binding site. The δ value reflects the fraction of membrane electric field that the charged blocking molecule crosses on its pathway between the external media and the binding site in the channel.

Our experimental data were not well fitted by abovementioned equation, presumably because of the presence of voltage-independent component. Thus, we deduced Equation (2), taking it into account and assuming that the binding to the deep and shallow site is independent:

(2) B=100−100/(1+C/(Kvd∗exp⁡〖Fzδ/RTV〗)+C/Kvi+(C∗C)/((Kvd∗exp⁡〖Fzδ/RTV〗∗Kvi))

In this equation, Kvd is the affinity of the drug to the deep site, Kvi—the affinity of the drug to the shallow site, and other parameters are the same as in Equation (1).

### 4.4. Statistical Analysis

All experimental data are presented as the mean ± SD estimated from at least four experiments (cells). Significance of the effects was tested with *t*-tests. Differences were considered significant at *p* < 0.05. Concentration dependencies were approximated with Hill equation. Voltage dependencies were approximated with Equation (2). Patch destabilization with a large number of transitions between different potential states and the limited capability of the application system (a maximum of 8 solutions) did not allow for an estimation of the entire voltage dependence and agonist dependence, respectively, in a single experiment. The data from different cells (*n* ≥ 4 for each holding potential or NMDA concentration value) were pooled together and fitted with Equation (2) (voltage dependence) or Hill equation (agonist dependence) using Origin 2019b 9.65 (OriginLab Corp., Northampton, MA, USA) software. Approximation error values were taken as the precision measures.

## Figures and Tables

**Table 1:** Quantitative characteristics of NMDA receptor inhibition by sepimostat, nafamostat, and diarylamidine compounds.

Compound	Chemical Structure	IC_50_, µM −80 mV	Hill Coeff.	K_vi_, µM	K_vd_, µM	zδ
Sepimostat	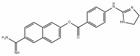	3.5 ± 0.3	0.9 ± 0.1	7 ± 1 7 ± 1 *	~130 102 ± 24 *	0.6 ± 0.2 0.55 *
Nafamostat	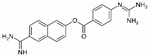	0.20 ± 0.04	1.2 ± 0.2	2.9 ± 0.5 3.0 ± 0.4 *	12 ± 3 10.5 ± 0.7 *	1.1 ± 0.1 1.1 *
Furamidine	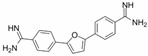	0.64 ± 0.13	1.5 ± 0.2	3.4 ± 0.4 3.3 ± 0.3 *	20 ± 5 21.8 ± 1.5 *	1.1 ± 0.1 1.1 *
Pentamidine	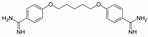	0.41 ± 0.08	1.1 ± 0.2	8 ± 1 10 ± 2 *	60 ± 25 27 ± 3 *	1.4 ± 0.1 1.1 *
Diminazene	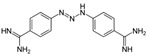	13 ± 3	2.0 ± 0.3	14 ± 2 14 ± 2 *	~600 1500 ± 500 *	0.9 ± 0.4 1.1 *
DAPI	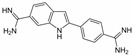	3.1 ± 0.6	1.2 ± 0.2	n.a. ** 3.16 ± 0.01 *	n.a. 5000 ± 100 *	0.06 ± 0.70 1.1 *
Gabexate	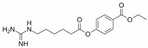	16 ± 3	0.8 ± 0.2	29 ± 8 25 ± 2 *	~300 800 ± 200 *	0.4 ± 0.2 0.55 *
Camostat	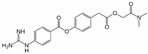	>100	n.a.	n.a.	n.a.	n.a.

* binding constants calculated using Equation (2) with fixed zδ values of 0.55 and 1.1 for mono- and dicationic compounds, respectively. Sepimostat was considered as monocation. ** not applicable.

## Data Availability

The data presented in this study are available on request from the corresponding author.

## References

[1] Hansen K.B., Yi F., Perszyk R.E., Furukawa H., Wollmuth L.P., Gibb A.J., Traynelis S.F. (2018). Structure, function, and allosteric modulation of NMDA receptors. J. Gen. Physiol..

[2] Hansen K.B., Wollmuth L.P., Bowie D., Furukawa H., Menniti F.S., Sobolevsky A.I., Swanson G.T., Swanger S.A., Greger I.H., Nakagawa T. (2021). Structure, Function, and Pharmacology of Glutamate Receptor Ion Channels. Pharmacol. Rev..

[3] Mayer M.L., Westbrook G.L., Guthrie P.B. (1984). Voltage-dependent block by Mg^2+^ of NMDA responses in spinal cord neurones. Nature.

[4] Nowak L., Bregestovski P., Ascher P., Herbet A., Prochiantz A. (1984). Magnesium gates glutamate-activated channels in mouse central neurones. Nature.

[5] Lipton S.A. (2006). Paradigm shift in neuroprotection by NMDA receptor blockade: Memantine and beyond. Nat. Rev. Drug Discov..

[6] Berman R.M., Cappiello A., Anand A., Oren D.A., Heninger G.R., Charney D.S., Krystal J.H. (2000). Antidepressant effects of ketamine in depressed patients. Biol. Psychiatry.

[7] Chaki S., Watanabe M. (2023). Antidepressants in the post-ketamine Era: Pharmacological approaches targeting the glutamatergic system. Neuropharmacology.

[8] Keam S.J. (2022). Dextromethorphan/Bupropion: First Approval. CNS Drugs.

[9] Szasz B.K., Mike A., Karoly R., Gerevich Z., Illes P., Vizi E.S., Kiss J.P. (2007). Direct inhibitory effect of fluoxetine on N-methyl-D-aspartate receptors in the central nervous system. Biol. Psychiatry.

[10] Barygin O.I., Nagaeva E.I., Tikhonov D.B., Belinskaya D.A., Vanchakova N.P., Shestakova N.N. (2017). Inhibition of the NMDA and AMPA receptor channels by antidepressants and antipsychotics. Brain Res..

[11] Adolph O., Koster S., Georgieff M., Georgieff E.M., Moulig W., Fohr K.J. (2012). Promethazine inhibits NMDA-induced currents—New pharmacological aspects of an old drug. Neuropharmacology.

[12] Fohr K.J., Zeller K., Georgieff M., Koster S., Adolph O. (2015). Open channel block of NMDA receptors by diphenhydramine. Neuropharmacology.

[13] Sugimoto M., Uchida I., Mashimo T. (2003). Local anaesthetics have different mechanisms and sites of action at the recombinant N-methyl-D-aspartate (NMDA) receptors. Br. J. Pharmacol..

[14] Reynolds I.J., Aizenman E. (1992). Pentamidine is an N-methyl-D-aspartate receptor antagonist and is neuroprotective in vitro. J. Neurosci..

[15] Williams K., Dattilo M., Sabado T.N., Kashiwagi K., Igarashi K. (2003). Pharmacology of delta2 glutamate receptors: Effects of pentamidine and protons. J. Pharmacol. Exp. Ther..

[16] Dron M.Y., Zhigulin A.S., Barygin O.I. (2020). Mechanisms of NMDA receptor inhibition by diarylamidine compounds. Eur. J. Neurosci..

[17] Zhigulin A.S., Barygin O.I. (2022). Mechanisms of NMDA receptor inhibition by nafamostat, gabexate and furamidine. Eur. J. Pharmacol..

[18] Nakamura K., Johmura A., Oda M., Ino Y., Uchiyama H., Ohtani H., Miyazaki H., Kurumi M., Akizawa Y., Oka T. (1995). Inhibitory effects of sepimostat mesilate (FUT-187) on the activities of trypsin-like serine proteases in vitro. Yakugaku Zasshi.

[19] Iwaki M., Ino Y., Motoyoshi A., Ozeki M., Sato T., Kurumi M., Aoyama T. (1986). Pharmacological studies of FUT-175, nafamostat mesilate. V. Effects on the pancreatic enzymes and experimental acute pancreatitis in rats. Jpn. J. Pharmacol..

[20] Murakami H., Togawa M., Takahashi S., Kasahara N., Yamamoto J., Matsuura N., Koshiyama Y., Ino Y., Oda M. (1990). Pharmacological studies of 6-amidino-2-naphthyl 4-[(4,5-dihydro-1H-imidazol-2-yl)amino]benzoate dimethanesulfonate. Effects on experimental pancreatitis. Arzneimittelforschung.

[21] Fuwa M., Kageyama M., Ohashi K., Sasaoka M., Sato R., Tanaka M., Tashiro K. (2019). Nafamostat and sepimostat identified as novel neuroprotective agents via NR2B N-methyl-D-aspartate receptor antagonism using a rat retinal excitotoxicity model. Sci. Rep..

[22] Sobolevsky A.I., Koshelev S.G., Khodorov B.I. (1999). Probing of NMDA channels with fast blockers. J. Neurosci..

[23] Blanpied T.A., Boeckman F.A., Aizenman E., Johnson J.W. (1997). Trapping channel block of NMDA-activated responses by amantadine and memantine. J. Neurophysiol..

[24] Bolshakov K.V., Gmiro V.E., Tikhonov D.B., Magazanik L.G. (2003). Determinants of trapping block of N-methyl-d-aspartate receptor channels. J. Neurochem..

[25] Kotermanski S.E., Johnson J.W. (2009). Mg^2+^ imparts NMDA receptor subtype selectivity to the Alzheimer’s drug memantine. J. Neurosci..

[26] Nikolaev M.V., Magazanik L.G., Tikhonov D.B. (2012). Influence of external magnesium ions on the NMDA receptor channel block by different types of organic cations. Neuropharmacology.

[27] Foster K.A., McLaughlin N., Edbauer D., Phillips M., Bolton A., Constantine-Paton M., Sheng M. (2010). Distinct roles of NR2A and NR2B cytoplasmic tails in long-term potentiation. J. Neurosci..

[28] Karlov D.S., Temnyakova N.S., Vasilenko D.A., Barygin O.I., Dron M.Y., Zhigulin A.S., Averina E.B., Grishin Y.K., Grigoriev V.V., Gabrel’yan A.V. (2022). Biphenyl scaffold for the design of NMDA-receptor negative modulators: Molecular modeling, synthesis, and biological activity. RSC Med. Chem..

[29] Song X., Jensen M.O., Jogini V., Stein R.A., Lee C.H., McHaourab H.S., Shaw D.E., Gouaux E. (2018). Mechanism of NMDA receptor channel block by MK-801 and memantine. Nature.

[30] Chou T.H., Epstein M., Michalski K., Fine E., Biggin P.C., Furukawa H. (2022). Structural insights into binding of therapeutic channel blockers in NMDA receptors. Nat. Struct. Mol. Biol..

[31] Antonov S.M., Johnson J.W. (1996). Voltage-dependent interaction of open-channel blocking molecules with gating of NMDA receptors in rat cortical neurons. J. Physiol..

[32] Vorobjev V.S. (1991). Vibrodissociation of sliced mammalian nervous tissue. J. Neurosci. Methods.

[33] Jun S.B., Cuzon Carlson V., Ikeda S., Lovinger D. (2011). Vibrodissociation of neurons from rodent brain slices to study synaptic transmission and image presynaptic terminals. J. Vis. Exp..

[34] Magazanik L.G., Buldakova S.L., Samoilova M.V., Gmiro V.E., Mellor I.R., Usherwood P.N. (1997). Block of open channels of recombinant AMPA receptors and native AMPA/kainate receptors by adamantane derivatives. J. Physiol..

[35] Woodhull A.M. (1973). Ionic blockage of sodium channels in nerve. J. Gen. Physiol..

